# Protective Effects of Higher Exposure to Aspirin and/or Clopidogrel on the Occurrence of Hip Fracture among Diabetic Patients: A Retrospective Cohort Study

**DOI:** 10.3390/biomedicines10102626

**Published:** 2022-10-19

**Authors:** Jui-Ting Mao, Jung-Nien Lai, Yi-Hsiu Fu, Hei-Tung Yip, Yen-Chun Lai, Chung-Y. Hsu, Sung-Hsiung Chen, Shu-Jui Kuo

**Affiliations:** 1 Department of Orthopedic Surgery, China Medical University Hospital, Taichung 404, Taiwan; 2 Department of Chinese Medicine, China Medical University Hospital, Taichung 404, Taiwan; 3 School of Chinese Medicine, China Medical University, Taichung 404, Taiwan; 4 Department of Education, Taichung Veterans General Hospital, Taichung 407, Taiwan; 5 Management office for Health Data, China Medical University Hospital, Taichung 404, Taiwan; 6 School of Medicine, National Taiwan University, Taipei 100, Taiwan; 7 Graduate Institute of Biomedical Sciences, China Medical University, Taichung 404, Taiwan; 8 Department of Orthopedic Surgery, College of Medicine, Chang Gung University, Kaohsiung Chang Gung Memorial Hospital, Kaohsiung 833, Taiwan; 9 School of Medicine, China Medical University, Taichung 404, Taiwan

**Keywords:** aspirin, clopidogrel, hip fracture, type 2 diabetes

## Abstract

Aspirin and clopidogrel are commonly prescribed alone or together among the type 2 diabetes mellitus (T2DM) patients, and both agents could affect bone metabolism. This study aimed at demonstrating the effects of the dosage and the duration of aspirin and/or clopidogrel alone or together on the occurrence of hip fracture among T2DM patients. We chose the patients newly diagnosed with T2DM and divided them into four subgroups which are under aspirin monotherapy (78,522 patients), clopidogrel monotherapy (12,752 patients), dual therapy (7209 patients), and patients not taking antiplatelet drugs (401,686 patients). We found that only higher dosage (>360 cumulative daily defined dose (cDDD)) and longer duration (≥3 years) of antiplatelet agents could be associated with lower fracture risk. Compared with the subjects taking <1-year dual agents, the risk of hip fracture was 0.38-fold for the patients taking ≥3-year dual agents. Lower dosage (28–179 cDDD) and shorter duration (1~2 years) could even be associated with higher fracture risk. Overall, the best regimen to fend off the hip fracture was the use of aspirin and clopidogrel for ≥3 years.

## 1. Introduction

The rising prevalence of type 2 diabetes mellitus (T2DM) is an important public health issue [1]. The number of DM patients worldwide has doubled in the past thirty years. T2DM is associated with higher risk of thromboembolic diseases, such as coronary artery disease and cerebrovascular disease. The risk of hip fracture is also higher among T2DM patients [2]. Thromboembolic diseases and hip fracture are potentially fatal, and the therapies that can treat and prevent these potentially fatal morbidities among T2DM patients are a major field worthy of discussion.

Antiplatelet drugs are widely used in the treatment and prevention of thromboembolic diseases among T2DM patients. Aspirin is an inhibitor of prostaglandin synthesis and may impact bone remodeling [3]. Clopidogrel is a thienopyridine and inhibits platelet function by irreversibly inhibiting the P2Y12 adenosine diphosphate (ADP) receptor, which are involved in the regulation of metabolic activities of bone cells [4,5]. Aspirin and clopidogrel are commonly prescribed alone or together for T2DM patients, and, interestingly, both agents could affect bone metabolism. It is thus an interesting issue that whether these two agents could affect the risk of hip fracture, another T2DM-related fatal morbidity other than thromboembolic diseases, among T2DM patients.

Osteoporosis is a prevalent disease in the aging society, and the gravest complication associated with osteoporosis is hip fracture. Hip fractures account for one-fourth of geriatric fractures necessitating hospitalization with the mortality rate reaching 20% [6,7,8,9,10]. T2DM patients are prone to suffer from higher risk of hip fracture, death after fracture, and hip fracture-free mortality. T2DM males and females are at 28% and 57% excessive risk of mortality after suffering from hip fracture, respectively [2]. In fact, the impact of aspirin and clopidogrel, as a single agent, on the occurrence of hip fracture for the general population has been discussed in the previous literature, and the results vary substantially across different studies due to coarse stratification of dosage and duration [11]. The effects of combination of the two agents on fracture risk have not been explored.

In view of the limitations of current publications, we conducted a population-based study to investigate the impact of aspirin and/or clopidogrel with detailed stratification of dosage and duration on the risk of hip fracture among T2DM patients. The effects of combination therapy were also determined. We also investigated the risk of hemorrhagic stroke among the T2DM patients under antiplatelet therapy. We hypothesize that antiplatelet agents could affect the risk of hip fracture among T2DM patients.

## 2. Materials and Methods

### 2.1. Data Source

Taiwan National Health Insurance Research Database (NHIRD) contains medical data of around 23 million residents. We utilized the dataset of Longitudinal Cohort of Diabetes Patients for this cohort study. The dataset contains the medical record of inpatient/outpatient records of disease diagnosis, drug prescriptions or therapy, which was coded using the International Classification of Diseases Clinical Modification, 9th Revision (ICD-9 CM). This study was approved by the Institutional Review Board of China Medical University Hospital Research Ethics Committee (CMUH104-REC2-115(AR-4)). Patient consent was waived under the agreement of the Committee because the identity of the subjects could not be identified.

### 2.2. Patient Population

We selected the patients who were newly diagnosed with T2DM within 2000 to 2012 as the study population. These T2DM were then divided into antiplatelet users (including aspirin monotherapy, clopidogrel monotherapy, and dual therapy) and antiplatelet non-users. The first prescription date was the index date for the antiplatelet users and a random date between 2000 and 2012 was set as the index date for the non-users. The exclusion criteria were hip fracture occurred before the index date, missing information of sex, previous use of anti-resorptive or anabolic agents for osteoporosis, and age below 18. All the antiplatelet users were propensity-score matched by four T2DM non-users. Finally, there were 78,522 patients under aspirin monotherapy, 12,752 patients under clopidogrel monotherapy and 7209 patients under dual therapy. The 401,686 T2DM patients without the use of antiplatelet agents were assigned to control group.

### 2.3. Main Outcome and Covariates

The risk of hip fracture (ICD-9-CM code 820, A473) was the outcome of interest in our study. Comorbidities included hypertension (ICD-9-CM code 401–405, A260, A269), hyperlipidemia (ICD-9-CM code 272), coronary artery disease (CAD) (ICD-9-CM code 410–414, A270, A279), stroke (ICD-9-CM code 430–438), arrhythmia (ICD-9-CM code 427), chronic kidney disease (CKD) (ICD-9-CM code 585), chronic obstructive pulmonary disease (COPD) (ICD-9-CM code 490–492, 496), and osteoporosis (ICD-9-CM code 733.0). Charlson Comorbidity Index (CCIs) and adapted Diabetes Complication Severity Index (aDSCI) were employed to evaluate the seriousness of diabetes mellitus patients. Antidiabetic drugs, antihypertensive drugs, statins, non-steroidal anti-inflammatory drug (NSAIDs), and oral steroids were also included as covariates in our model.

### 2.4. Statistical Analysis

The between-group differences were shown as standardized mean difference (SMD). The SMD method has been employed to estimate the similarity of baseline characteristics in propensity-score-matched cohorts. An SMD < 0.10 indicates a negligible imbalance between study individuals and their matched controls [12]. Cox proportional hazards models were applied to assess the hazard ratio (HR) and unravel the correlation between the exposure to antiplatelet agents and hip fracture [13,14]. A multivariate Cox proportional hazards model was utilized to assess the adjusted HRs (aHRs) after adjustment for age, sex, comorbidities, medications, CCIs, and aDCSI. An analysis of stratification by age, sex, CCIs, and aDCSI was implemented to assess the correlation between exposure to antiplatelet agents and hip fracture among the cohort of interest. Subhazard ratios (SHRs) were calculated employing competing risk regression models, considering death as the competing risk factor. The cumulative incidence of hip fracture was expressed by the Kaplan–Meier method, and a log-rank test was applied for the comparison of the incidence curves [12]. All statistical analyses were carried out applying STATA/SE software (version 14.0, STATA Corp., College Station, TX, USA). Two-tailed tests were applied to assess the statistical significance (*p* < 0.05).

## 3. Results

There were 78,522 patients under aspirin monotherapy, 12,752 patients under clopidogrel monotherapy, and 7209 patients under dual therapy. The 401,686 individuals without the use of antiplatelet agents were assigned to control group. The distributions of the comorbidities among the four groups were comparable under the propensity score matching. The score of baseline CCI and baseline aDSCI as well as the medicine taken by the participants were also comparable between the four group (all SMD < 0.10) (Table 1).

**Figure 1:**
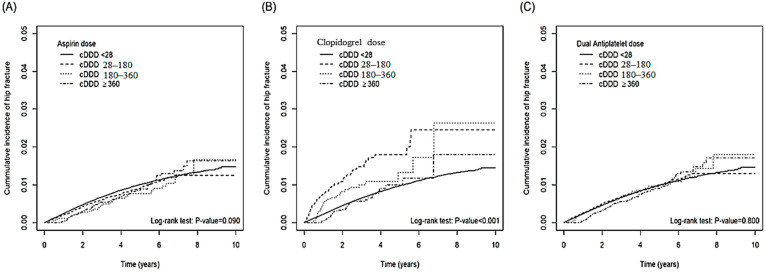
The cumulative incidence of hip fracture among patients with different cumulative defined daily doses (cDDDs) of aspirin (**A**), clopidogrel (**B**), and dual anti-platelet agents (**C**).

**Figure 2:**
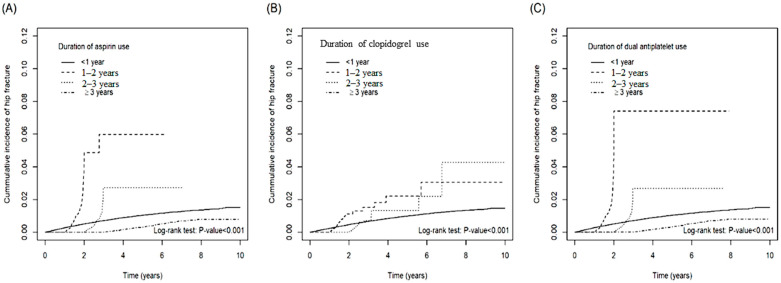
The cumulative incidence of hip fracture among patients with different duration (years) of aspirin (**A**), clopidogrel (**B**), and dual anti-platelet agents (**C**).

Table 2 demonstrates the incidence of hip fracture stratified by dosage and duration of the use of antiplatelet agents. The risk of hip fracture among the patients taking 28–179 cumulative daily defined dose (cDDD) aspirin was 1.13-fold (aHR: 1.01~1.25) than that of the patients taking <28 cDDD aspirin. Compared with the subjects taking <28 cDDD clopidogrel, the risk of hip fracture was 1.39-fold (aHR: 1.10~1.75) and 0.51-fold (aHR: 0.37~0.72) for the patients taking 28–179 cDDD and ≥360 cDDD clopidogrel, respectively. Compared with the subjects taking< 28 cDDD dual agents, the risk of hip fracture was 1.18-fold (aHR: 1.06~1.30) and 0.74-fold (aHR: 0.61~0.91) for the patients taking 28–179 cDDD and ≥360 cDDD dual agents, respectively. Compared with the subjects taking <1-year aspirin, the risk of hip fracture was 2.54-fold (aHR: 2.14~3.02) and 0.42-fold (aHR: 0.35~0.49) for the patients taking 1–2 year and ≥3-year aspirin, respectively. Compared with the subjects taking <1-year clopidogrel, the risk of hip fracture was 0.52-fold (aHR: 0.33~0.80) for the patients taking 2–3-year clopidogrel. Compared with the subjects taking< 1-year dual agents, the risk of hip fracture was 1.97-fold (aHR: 1.68~2.30) and 0.38-fold (aHR: 0.33~0.45) for the patients taking 1–2 year and ≥3-year dual agents, respectively. The cumulative incidence curves of hip fracture stratified by cDDD and duration for the patients taking aspirin, clopidogrel, and dual agents were illustrated in Figure 1 and Figure 2. The cumulative incidence of hip fracture varied significantly among different cDDD-based stratification in the aspirin monotherapy group (*p* < 0.001 by log rank test). The cumulative incidence of hip fracture also varied significantly among different duration-based stratification in the aspirin monotherapy group (*p* < 0.001 by log rank test), clopidogreal monotherapy group (*p* < 0.001 by log rank test), and dual therapy group (*p* < 0.001 by log rank test). The fracture risk reduction effects of antiplatelet agents are only present in the higher cDDD and longer duration curves. The Table 2, Figure 1 and Figure 2 showed that antiplatelet agents could only exert protective effects in higher dosage (≥360 cDDD) and longer duration (≥3-year) ranges. Lower dosage (28–179 cDDD) and shorter duration (1~2 years) could even be associated with higher risk of hip fracture.

Table 3 shows the risk of hip fracture stratified by sex, age, CCIs, aDCSI, and the diagnosis of osteoporosis. In general, the impact of antiplatelet agents on hip fracture could not be demonstrated without the stratification of dosage and duration. However, the stratification based on age, CCIs, aDCSI, and the diagnosis of osteoporosis showed that the female T2DM patients undergoing aspirin monotherapy seems to have lower risk of hip fracture than the female T2DM patients without any anti-platelet therapy.

After considering death as a competing event, the trend that antiplatelet agents could only exert protective effects against fracture in higher exposure range is similar. Overall, the patients taking dual antiplatelet agents had 0.59-fold (aSHR: 0.42~0.82) the risk of fracture than the non-users. Patients taking 180–359 cDDD clopidogrel (not 28–179 cDDD) had 0.21-fold (aSHR: 0.07~0.66) the risk of fracture than the non-users. Compared with the subjects taking <1-year aspirin, the risk of hip fracture was 8.33-fold (aSHR: 5.31~13.08), 5.09-fold (aSHR: 3.41~7.61), and 0.54-fold (aSHR: 0.44~0.68) for the patients taking 1–2 years, 2–3 years, and ≥3-years aspirin, respectively. Patients taking 1–2 year clopidogrel had 2.06-fold (aSHR: 1.15~3.68) the risk of fracture than the non-users. Compared with the subjects taking< 1-year dual agents, the risk of hip fracture was 8.07-fold (aSHR: 5.15~12.64), 4.89-fold (aSHR: 3.30~7.24), and 0.50-fold (aSHR: 0.40~0.62) for the patients taking 1–2 years, 2–3 years, and ≥3-years dual agents, respectively (Table 4).

Table 5 demonstrates the risk of hemorrhagic stroke stratified by dosage and duration of the use of antiplatelet agents. Antiplatelet agents did not increase the risk of hemorrhagic stroke in any range of dosage and duration.

## 4. Discussion

In our study, we showed that lower exposure (28–179 cDDD, 1–2 years) to antiplatelet agents could not exert protective effects (or even detrimental) to the hip fracture. The best regimen to fend off the hip fracture was the use of aspirin and clopidogrel for ≥3 years. As for the risk of major bleeding, antiplatelet agents did not increase the risk of hemorrhagic stroke in any range of dosage and duration. These results have not been published before and merit noticing.

Aspirin has multiple mechanisms of action including effects on prostaglandin production and nitric oxide (NO) and NF-κB production, all of them may affect bone metabolism [15]. The mechanism by which aspirin facilitates bone anabolism is through irreversible inhibition of cyclooxygenase (COX) [16]. Bauer et al. evaluated the risk factors for osteoporosis and the exposure to aspirin and NSAIDs in 7786 Caucasian women over 65 years of age for 4 years. The authors showed that regular administration of aspirin or NSAIDs may exert mild increments in bone mineral density (BMD) among postmenopausal females. This phenomenon persists after adjustment for the presence of osteoarthritis and obesity. However, among the females who take aspirin or NSAIDs regularly, there is no significant risk-reduction effects on the subsequent fractures [17]. In another study, NSAID and aspirin use and BMD were assessed in 2853 adults (49.5% women, 50.5% men; mean age: 73.6 years). After adjustment for pertinent confounders, current exposure to COX-2 selective NSAIDs with aspirin was correlated with higher BMD at the whole body (4.2%), and total hip (4.6%) by dual-energy X-ray absorptiometry and at both cortical spine (12.8%) and trabecular (34.1%) by quantitative computed tomography. The authors thus concluded that the combination of relative COX-2 selective NSAIDs and aspirin is associated with higher BMD at multiple skeletal sites in men and women [15]. These studies suggested the potential protective effects of aspirin [15,17]. On the contrary, one Danish study showed that low-dose aspirin is correlated with higher susceptibility to overall fractures and hip fractures, and clopidogrel is not correlated with increased fracture risk [18]. The authors tried to explain these seemly conflicting findings by the fact that this study only focused on low doses of aspirin prescribed for the prophylaxis of thromboembolic complications while the other studies focus on all dose ranges. Differences might also be present in the cohorts analyzed, especially as low dose and high dose (≥500 mg/day) aspirin are prescribed for different situations and thus the health conditions of the individuals enrolled might be of great heterogeneity. These findings suggested the importance of exposure stratification and population specification. The current publications discussing the correlation between aspirin and fracture are summarized in Table 6.

Clopidogrel is a thienopyridine and could irreversibly inhibit the P2Y12 ADP receptor [5]. Both osteoblast and osteoclast possess P2Y12 ADP receptor. Xinming Su et. al. showed that mice lacking P2Y12 ADP receptor had decreased osteoclast activity, and P2Y12 knock-out mice were partially protected from pathologic osteolysis associated with increased extracellular ADP [22]. However, clopidogrel also slowed proliferation and viability of osteoblasts [23]. Since clopidogrel inhibits both osteoblast and osteoclast, its final impact on bone metabolism would depend on the differential inhibitory extent. Jørgensen et al. investigated the association between clopidogrel use and fracture incidence in a Danish nationwide cohort study. All patients prescribed with clopidogrel during 1996–2008 were included (*n* = 77,503), and 3 nonusers were randomly matched for age and sex (*n* = 232,510) to each clopidogrel-treated subject. Treatment with clopidogrel was associated with increased overall and osteoporotic fracture risk, especially in subjects with a treatment duration > 1 year. However, individuals with low exposure to clopidogrel (<0.01 defined daily dose) had a lower risk of fracture than never users. The authors thus conclude that use of clopidogrel is associated with risk of fractures [4]. The same author group narrowed down the population to stroke patients. After adjusting for multiple confounders, the exposure to clopidogrel was not correlated with increased fracture risk among individuals with transient ischemic attack (TIA) or ischemic stroke. However, after adjusting for pertinent confounders, the exposure to clopidogrel was correlated with a 10–35% reduction in fracture risk [24]. The publications from the same author group exhibited seemly contradictory results highlight the importance of sample population specification and the stratification based upon exposure (Table 7). The decreased and increased fracture risk among patients with high and low exposure to clopidogrel might be explained by the differential extent of suppression to osteoblast and osteoclast in differential exposure level to clopidogrel.

The inconclusive findings from previous publications may be explained by the different population and different exposure stratification. In view of the unaddressed issues of previous works, our study narrowed down our sample population to T2DM patients, and rigorous stratification based upon dose and duration was performed. Moreover, we specify the dual agent using population from the single agent using population following the analytic protocol of our previous works [12]. These endeavors substantially supplement our understanding about the impact of antiplatelet agents on bone health among T2DM patients.

There are some limitations in our study. First, the study is retrospective in nature and randomized control trial may be needed in the future. However, aspirin and clopidogrel are one of the standard treatments for several chronic disease with proven benefits, so it would have ethical problem to include controlled group in population. Second, despite many confounding factors were adjusted in our study, there were still other confounding factors not included that may influence the results, such as the serum 25-(OH)-D level and bone densiometric findings. In other words, it is impossible to include all the confounders in our model. Moreover, our study was performed in the Taiwanese ethnic group, and these findings should be extrapolated to other ethnic groups (such as Caucasians) with due caution. To draw a general conclusion about the impacts of antiplatelet agents on bone health without the overall fractures data indeed limits the validity and generalizability of the conclusions. The reason we investigate hip fracture only is because some osteoporotic fractures, such as proximal humerus fractures and distal radius fractures, might not be accurately coded if the patients did not chose surgical treatment.

However, our study bears some strengths which deserve noticing. We clearly offered the rigorous stratification of the duration and dosage of aspirin, clopidogrel, and dual agents, demonstrating the differential association between the risk of hip fracture and the exposure of antiplatelet agents. We highlighted the group of combined aspirin and clopidogrel, demonstrating that the stratification associated with the lowest risk of hip fracture was the use of aspirin and clopidogrel for ≥3 years. We also investigated the risk of hemorrhagic stroke among T2DM patients under antiplatelet therapy. These findings help clinicians balance between the risk of hip fracture and the risk of bleeding-related morbidities when prescribing antiplatelet agents.

## 5. Conclusions

According to our study, only higher dosage (>360 cDDD) as well as longer duration of (≥3 years) antiplatelet agents could be associated with lower risk of hip fracture. Lower dosage (28–179 cDDD) and shorter duration (1~2 years) could even be associated with higher fracture risk.

## Figures and Tables

**Table 1:** Baseline characteristics of the four cohorts.

	Antiplatelet Non-User (*N* = 401,686)		Antiplatelet Users (*N* = 98,483)						
			Aspirin Monotherapy		Clopidogrel Mono Therapy		Dual Therapy		
			*N* = 78,522		*N* = 12,752		*N* = 7209		
Variables	*n*	%	*n*	%	*n*	%	*n*	%	SMD
Sex									0.035
Female	192,615	48%	39,691	51%	3766	30%	2056	29%	
Male	209,071	52%	38,831	49%	8986	70%	5153	71%	
Age, year									0.064
<30	9487	2.4%	1850	2.4%	16	0.1%	13	0.2%	
30–50	95,811	24%	20,986	27%	1574	12%	1241	17%	
50–70	217,461	54%	44,284	56%	6981	55%	4176	58%	
≥70	78,927	20%	11,402	15%	4181	33%	1779	25%	
Mean (SD)	58.1	(13.6)	56.4	(12.6)	64.1	(12.1)	61.2	(12.0)	0.025
Comorbidities									
Hypertension	258,897	64%	46,129	59%	10,484	82%	5314	74%	0.033
Hyperlipidemia	258,217	64%	48,601	62%	8729	68%	4578	64%	0.030
CAD	104,214	26%	15,162	19%	6503	51%	2556	35%	0.031
Stroke	5194	1%	617	1%	694	5%	161	2%	0.017
Arrhythmia	54,806	14%	8965	11%	2669	21%	1136	16%	0.020
CKD	15,657	4%	2317	3%	1400	11%	529	7%	0.021
COPD	77,572	19%	14,062	18%	3162	25%	1570	22%	0.006
Osteoporosis	2162	1%	428	1%	62	0.5%	28	0.4%	0.002
CCIs									0.081
0	249,662	62%	51,785	66%	3162	25%	2873	40%	
1	61,021	15%	11,571	15%	3851	30%	2042	28%	
2	46,872	12%	8194	10%	2327	18%	1033	14%	
≥3	44,131	11%	6972	9%	3412	27%	1261	17%	
aDCSI									0.049
0	333,706	83%	66,690	85%	8018	63%	5502	76%	
1	34,738	9%	7112	9%	1943	15%	794	11%	
≥2	33,242	8%	4720	6%	2791	22%	913	13%	
Medications									
Anti-DM drugs									
Metformin	213,265	53%	40,974	52%	7915	62%	4278	59%	0.018
Sulfonylureas	184,031	46%	35,903	46%	6960	55%	3956	55%	0.035
Alpha-glucosidase inhibitors	39,287	10%	7361	9%	1973	15%	798	11%	0.017
Thiazolidinedione	35,793	9%	7027	9%	1658	13%	752	10%	0.023
Insulin	37,023	9%	6761	9%	2573	20%	1258	17%	0.051
Anti-HTN drugs									
α-blockers	73,503	18%	12,196	16%	4045	32%	1839	26%	0.002
β-blockers	211,920	53%	37,258	47%	8615	68%	4192	58%	0.038
Potassium-sparing diuretics	18,825	5%	3285	4%	1065	8%	496	7%	0.011
Thiazides diuretics	35,793	9%	7027	9%	1658	13%	752	10%	0.023
Loop diuretics	77,655	19%	13,436	17%	3857	30%	1818	25%	0.002
CCBs	212,199	53%	36,608	47%	9132	72%	4552	63%	0.035
ACEIs	183,404	46%	30,943	39%	8462	66%	4073	56%	0.030
ARBs	88,513	22%	14,865	19%	4590	36%	2044	28%	0.005
Statins	161,715	40%	28,131	36%	7097	56%	3155	44%	0.026
NSAIDs	389,629	97%	76,153	97%	12,095	95%	6827	95%	0.026
Oral steroid	312,091	78%	61,695	79%	9180	72%	5078	70%	0.014

SMD: standard mean difference; CAD: coronary artery disease; CKD: chronic kidney disease; COPD: chronic obstructive pulmonary disease; HTN: hypertension; CCIs: Charlton comorbidity index; aDCSI: adapted diabetes complication severity index; CCBs: calcium channel blockers; ACEIs: angiotensin-converting enzyme inhibitors; ARBs: angiotensin II receptor blockers; NSAIDs: non-steroidal anti-inflammatory drug.

**Table 2:** Hazard ratio of hip fracture stratified by dosage and duration of antiplatelet agents.

	Hip Fracture								
Variables	*n*	PY	IR	cHR	(95% CI)	*p*-Value	aHR	(95% CI)	*p*-Value
Aspirin or clopidogrel									
Non-users	2558	1,272,862	2.01	1.00	(reference)		1.00	(reference)	
Users	638	313,212	2.04	1.01	(0.92, 1.1)	0.849	1.04	(0.96, 1.14)	0.338
Aspirin or clopidogrel									
Non-users	2558	1,272,862	2.01	1.00	(reference)		1.00	(reference)	
Aspirin Monotherapy	480	265,781	1.81	0.90	(0.82, 0.99)	0.035	1.08	(0.98, 1.20)	0.111
Clopidogrel Monotherapy	101	23,862	4.23	1.95	(1.60, 2.38)	<0.001	1.02	(0.83, 1.24)	0.866
Dual Therapy	57	23,568	2.42	1.19	(0.92, 1.55)	0.190	0.83	(0.63, 1.07)	0.152
Dosage of aspirin (cDDD)									
<28 cDDD	2683	1,305,766	2.05	1.00	(reference)		1.00	(reference)	
28–179 cDDD	395	210,415	1.88	0.91	(0.82, 1.02)	0.093	1.13	(1.01, 1.25)	0.027
180–359 cDDD	51	32,718	1.56	0.77	(0.58, 1.01)	0.063	0.84	(0.63, 1.10)	0.210
≥360 cDDD	67	37,175	1.80	0.90	(0.7, 1.14)	0.372	0.94	(0.74, 1.20)	0.613
Dosage of clopidogrel (cDDD)									
<28 cDDD	3049	1,542,881	1.98	1.00	(reference)		1.00	(reference)	
28–179 cDDD	76	14,792	5.14	2.49	(1.98, 3.12)	<0.001	1.39	(1.10, 1.75)	0.005
180–359 cDDD	37	10,991	3.37	1.61	(1.17, 2.23)	0.004	1.00	(0.73, 1.39)	0.979
≥360 cDDD	34	17,410	1.95	0.94	(0.67, 1.32)	0.734	0.51	(0.37, 0.72)	<0.001
Dosage of dual agents (cDDD)									
<28 cDDD	2556	1,272,011	2.01	1.00	(reference)		1.00	(reference)	
28–179 cDDD	450	217,019	2.07	1.03	(0.93, 1.13)	0.610	1.18	(1.06, 1.30)	0.002
180–359 cDDD	87	41,917	2.08	1.03	(0.83, 1.27)	0.814	0.92	(0.75, 1.15)	0.475
≥360 cDDD	103	55,127	1.87	0.93	(0.76, 1.13)	0.449	0.74	(0.61, 0.91)	0.003
Duration of aspirin (year)									
<1	2816	1,312,458	2.15	1.00	(reference)		1.00	(reference)	
1–2	143	21,487	6.66	2.87	(2.42, 3.41)	<0.001	2.54	(2.14, 3.02)	<0.001
2–3	92	36,480	2.52	1.08	(0.88, 1.33)	0.474	1.04	(0.84, 1.28)	0.739
≥3	145	215,649	0.67	0.32	(0.27, 0.38)	<0.001	0.42	(0.35, 0.49)	<0.001
Duration of clopidogrel (year)									
<1	3128	1,548,594	2.02	1.00	(reference)		1.00	(reference)	
1–2	37	9014	4.10	1.84	(1.33, 2.55)	<0.001	0.89	(0.64, 1.23)	0.464
2–3	20	9257	2.16	0.98	(0.63, 1.52)	0.918	0.52	(0.33, 0.80)	0.003
≥3	0	0	0.00	-	-				
Duration of dual agents (year)									
<1	2753	1,280,851	2.15	1.00	(reference)		1.00	(reference)	
1–2	176	27,714	6.35	2.75	(2.35, 3.22)	<0.001	1.97	(1.68, 2.30)	<0.001
2–3	109	43,826	2.49	1.06	(0.88, 1.29)	0.533	0.88	(0.72, 1.06)	0.185
≥3	158	233,683	0.68	0.32	(0.27, 0.38)	<0.001	0.38	(0.33, 0.45)	<0.001

*n*: number of event; PY: person-years; IR: incidence rate per 1000 person-years. cHR: crude hazard ratio; aHR: adjusted hazard ratio; CI: confidence interval; cDDD: cumulative defined daily dose; ꝉ: adjusted by sex, age, comorbidities, and medicine.

**Table 3:** Risk of hip fracture in different subgroups.

	Non-Users	Aspirin Monotherapy		Clopidogrel Monotherapy		Dual Antiplatelet Therapy	
Variables		aHR (95% CI)	*p*-Value	aHR (95% CI)	*p*-Value	aHR (95% CI)	*p*-Value
Sex							
Female	1.00	1.21 (1.06, 1.37)	0.004	1.03 (0.77, 1.39)	0.838	0.99 (0.68, 1.43)	0.958
Male	1.00	1.05 (0.81, 1.11)	0.522	1.03 (0.78, 1.35)	0.860	0.69 (0.48, 1.01)	0.057
Age							
<30	1.00	2.09 (0.74, 5.89)	0.165				
30–50	1.00	0.92 (0.65, 1.29)	0.614	1.08 (0.39, 2.96)	0.886	0.88 (0.32, 2.38)	0.798
50–70	1.00	1.07 (0.91, 1.24)	0.412	0.91 (0.61, 1.36)	0.657	0.95 (0.64, 1.42)	0.815
>70	1.00	1.02 (0.89, 1.17)	0.777	1.11 (0.87, 1.41)	0.390	0.70 (0.87, 1.41)	0.059
CCIs							
0	1.00	1.14 (0.99, 1.31)	0.065	0.84 (0.47, 1.49)	0.551	0.67 (0.38, 1.18)	0.165
1	1.00	0.97 (0.78, 1.22)	0.824	1.01 (0.66, 1.55)	0.952	1.08 (0.70, 1.65)	0.735
2	1.00	0.94 (0.73, 1.21)	0.629	0.98 (0.63, 1.52)	0.913	0.49 (0.23, 1.04)	0.064
≥3	1.00	1.10 (0.86, 1.40)	0.439	1.11 (0.82, 1.51)	0.498	0.84 (0.51, 1.39)	0.506
aDCSI							
0	1.00	1.08 (0.97, 1.20)	0.177	1.02 (0.80, 1.31)	0.853	0.77 (0.57, 1.05)	0.094
1	1.00	1.05 (0.75, 1.48)	0.770	0.80 (0.41, 1.57)	0.518	1.25 (0.62, 2.57)	0.521
≥2	1.00	1.17 (0.81, 1.69)	0.416	1.13 (0.74, 1.73)	0.577	0.85 (0.40, 1.80)	0.665
Osteoporosis							
No	1.00	1.05 (0.95, 1.16)	0.339	1.03 (0.84, 1.26)	0.781	0.82 (0.63, 1.07)	0.139
Yes	1.00	0.62 (0.23, 1.67)	0.344	0.84 (0.11, 6.38)	0.865		

cHR: crude hazard ratio; aHR: adjusted hazard ratio; 95% CI: 95% confidence interval; CCI: Charlson comorbidity index; aDSCI: adapted diabetes complication severity index.

**Table 4:** Incidence rate and subhazard ratio of hip fracture in patients using antiplatelet agents employing competing risks regression models.

Variables	cSHR (95% CI)	*p*-Value	aSHR (95% CI)	*p*-Value
Aspirin or clopidogrel				
Non-users	1.00 (reference)		1.00 (reference)	
users	1.67 (1.38, 2.03)	<0.001	0.90 (0.73, 1.11)	0.320
Aspirin or clopidogrel				
Non-users	1.00 (reference)		1.00 (reference)	
Aspirin Monotherapy	1.65 (1.34, 2.03)	<0.001	1.00 (0.81, 1.24)	0.960
Clopidogrel Monotherapy	2.68 (1.49, 4.80)	<0.001	0.96 (0.53, 1.74)	0.900
Dual Therapy	1.58 (1.14, 2.18)	0.010	0.59 (0.42, 0.82)	<0.001
Dose of aspirin				
<28 cDDD	1.00 (reference)		1.00 (reference)	
28–179 cDDD	1.41 (1.12, 1.78)	<0.001	0.95 (0.75, 1.21)	0.660
180–359 cDDD	1.58 (1.13, 2.21)	0.010	0.84 (0.60, 1.19)	0.330
>360 cDDD	1.70 (1.32, 2.19)	<0.001	0.88 (0.67, 1.15)	0.330
Dose of clopidogrel				
<28 cDDD	1.00 (reference)		1.00 (reference)	
28–179 cDDD	1.90 (1.26, 2.87)	<0.001	0.97 (0.64, 1.47)	0.890
180–359 cDDD	0.41 (0.13, 1.27)	0.120	0.21 (0.07, 0.66)	0.010
>360 cDDD	1.42 (0.93, 2.16)	0.100	0.67 (0.44, 1.03)	0.070
Dose of dual agents				
<28 cDDD	1.00 (reference)		1.00 (reference)	
28–179 cDDD	1.57 (1.24, 1.98)	<0.001	1.00 (0.79, 1.27)	0.960
180–359 cDDD	1.58 (1.11, 2.23)	0.010	0.80 (0.56, 1.14)	0.210
>360 cDDD	1.84 (1.45, 2.35)	<0.001	0.83 (0.64, 1.07)	0.150
Duration of aspirin (year)				
<1	1.00 (reference)		1.00 (reference)	
1–2	17.38 (11.13, 27.16)	<0.001	8.33 (5.31, 13.08)	0.000
2–3	10.62 (7.14, 15.81)	<0.001	5.09 (3.41, 7.61)	0.000
≥3	0.94 (0.76, 1.15)	0.530	0.54 (0.44, 0.68)	0.000
Duration of clopidogrel (year)				
<1	1.00 (reference)		1.00 (reference)	
1–2	3.67 (2.07, 6.52)	<0.001	2.06 (1.15, 3.68)	0.010
2–3	2.66 (1.37, 5.15)	<0.001	1.20 (0.61, 2.35)	0.610
Duration of dual agents (year)				
<1	1.00 (reference)		1.00 (reference)	
1–2	19.89 (12.81, 30.87)	<0.001	8.07 (5.15, 12.64)	<0.001
2–3	11.56 (7.88, 16.96)	<0.001	4.89 (3.30, 7.24)	<0.001
≥3	0.95 (0.77, 1.17)	0.630	0.50 (0.40, 0.62)	<0.001

cSHR: crude subhazard ratio; aSHR: adjusted subhazard ratio; 95% CI: 95% confidence interval; cDDD: cumulative daily defined dose.

**Table 5:** The hazard ratio of hemorrhagic stroke among patients taking antiplatelet agents.

	Hemorrhagic Stroke			
Variables	cHR (95% CI)	*p*-Value	aHR (95% CI)	*p*-Value
Aspirin or clopidogrel				
Non-users	1.00 (reference)		1.00 (reference)	
users	0.83 (0.45, 1.55)	0.558	0.8 (0.43, 1.49)	0.479
Aspirin or clopidogrel				
Non-users	1.00 (reference)		1.00 (reference)	
Aspirin monotherapy	0.57 (0.26, 1.25)	0.162	0.63 (0.29, 1.38)	0.250
Clopidogrel monotherapy	1.83 (0.44, 7.5)	0.404	0.91 (0.22, 3.81)	0.903
Dual Therapy	2.72 (0.85, 8.68)	0.091	1.74 (0.54, 5.62)	0.353
Dose of aspirin				
<28 cDDD	1.00 (reference)		1.00 (reference)	
28–179 cDDD	0.80 (0.38, 1.68)	0.561	0.92 (0.44, 1.93)	0.828
180–359 cDDD	0.65 (0.09, 4.68)	0.668	0.67 (0.09, 4.85)	0.692
>360 cDDD				
Dose of clopidogrel				
<28 cDDD	1.00 (reference)		1.00 (reference)	
28–179 cDDD	3.22 (0.79, 13.16)	0.103	1.74 (0.42, 7.18)	0.446
180–359 cDDD	4.30 (1.05, 17.61)	0.042	2.61 (0.63, 10.82)	0.187
>360 cDDD				
Dose of dual agents				
<28 cDDD	1.00 (reference)		1.00 (reference)	
28–179 cDDD	0.80 (0.38, 1.68)	0.559	0.86 (0.41, 1.8)	0.688
180–359 cDDD	2.08 (0.76, 5.74)	0.155	1.81 (0.66, 5)	0.253
>360 cDDD				
Duration of aspirin (year)				
<1	1.00 (reference)		1.00 (reference)	
1–2	0.50 (0.07, 3.58)	0.488	0.55 (0.08, 3.95)	0.550
≥2	0.65 (0.2, 2.06)	0.461	0.78 (0.24, 2.48)	0.669
Duration of clopidogrel (year)				
<1	1.00 (reference)		1.00 (reference)	
1–2				
≥2				
Duration of dual agents (year)				
<1	1.00 (reference)		1.00 (reference)	
1–2	0.42 (0.06, 3.04)	0.392	0.39 (0.05, 2.83)	0.353
≥2	0.53 (0.17, 1.7)	0.290	0.54 (0.17, 1.73)	0.303

CI: 95% confidence interval; cDDD: cumulative daily defined dose.

**Table 6:** Characteristics of publications discussing the correlation between aspirin and fracture.

	Design	Population Characteristics	Aspirin Exposure	Outcome
Bauer et al. (1996) [17]	Cohort	7786 US white women ≥ age 65	<1, 1–4, 5–7 use/week for 3.9 years	BMD In age-adjusted analyses, daily use of aspirin or NSAIDs was associated with 2.3–5.8% increase in BMD of the hip and spine. The relationship persisted even after adjustment for weight, medications, and self-reported and radiographic OA (adjusted increase in BMD: 1.0–3.1%). Fracture Fracture risk was similar among daily users of aspirin and NSAIDs and nonusers. After adjustment for confounders, among daily aspirin users the relative risk of hip fracture was 1.1 (95% CI: 0.7, 1.6), and among daily NSAID users the risk was 0.9 (CI: 0.6, 1.4). The risk among aspirin users was 1.0 (CI: 0.8, 1.2), and among NSAID users the risk was also 1.0 (CI: 0.8, 1.2) for all non-spine fractures.
Carbone et al. (2003) [15]	CS	70–79 years of age, no difficulty in walking one-quarter mile, climbing 10 stairs without resting. A total of 3075 subjects were enrolled from Memphis, Tennessee (*n* = 1548), and Pittsburgh, Pennsylvania (*n* = 1527).	328 ± 302 mg/day	BMD After adjustment for confounders, current use of COX-2 NSAIDs with aspirin was associated with higher BMD at the whole body (4.2%, 1.2–7.3 CI) and total hip (4.6%, 0.5–8.8 CI) by DXA and at both trabecular (34.1%, 15.4–52.7 CI) and cortical spine (12.8%, 2.3–23.3 CI) by qCT.
Vestergaard et al. (2006) [19]	CC	Danish population-based study, recruiting 124,655 fractures in year 2000. For each case, 3 controls (*n* = 373,962) matched on age and sex were randomly drawn from the background population.	<20, 20–74, >75 cDDD	Fracture For acetaminophen, a small increase in overall fracture risk was observed with use within the last year (OR = 1.45, 95% CI: 1.41–1.49). For aspirin, no increase in overall fracture risk was present with recent use. Significant heterogeneity was present for the NSAIDs; e.g., ibuprofen was associated with an increased overall fracture risk (OR = 2.09, 95% CI: 2.00–2.18 for <20 cDDD), while celecoxib was not (OR = 0.76, 95% CI: 0.51–1.13 for <20 cDDD). Osteoarthritis was associated with a decreased risk of any fracture if diagnosed > 1 year ago (OR = 0.70, 95% CI: 0.67–0.72).
Hill et al. (2008) [20]	CS	2501 men aged 40 to 93 years were recruited from the Caribbean Island of Tobago.	Self-report of use ≥ 3 times per week.	BMD *BMD* was 10% and 20% higher in African Caribbean males compared to U.S. non-Hispanic black and white males, respectively. Greater lean mass, history of working on a fishing boat or on a farm, frequent walking, and self-reported diabetes were associated with higher BMD. Fat mass, history of farming, and self-reported hypertension were associated with higher BMAD. Older age, mixed African ancestry, and history of a fracture were associated with lower BMD and BMAD. Lean body mass explained 20%, 18%, and 6% of the variance in BMD at the total hip, femoral neck, and BMAD, respectively.
Vestergaard et al. (2012) [18]	CC	Danish population-based study, recruiting 124,655 fractures in 2000.	≤150 mg/day	Fracture Use of low-dose aspirin was actually associated to a small decrease in fracture risk (OR = 0.93, 95% CI: 0.91–0.96).
Bonten et al. (2017) [21]	CS	Between 2008 and 2012, information on medication use and DXA measured vertebral and femoral BMD of 916 participants was collected in the Netherland Epidemiology of Obesity study. A subgroup analysis in postmenopausal women (*n* = 329) was conducted	30–125 mg for ≥1 year	BMD After adjustment, there was no difference between aspirin users and non-users for vertebral BMD (adjusted mean difference: 0.036 (95% CI −0.027 to 0.100) g/cm^2^) and femoral BMD (adjusted mean difference: 0.001 (−0.067 to 0.069) g/cm^2^). In the subgroup of postmenopausal women, aspirin use was not associated with lower vertebral (adjusted mean difference: 0.069 (−0.046 to 0.184) g/cm^2^) or femoral BMD (adjusted mean difference: −0.055 (−0.139; 0.029) g/cm^2^).

CC: case-control; CS: cross-sectional; BMD: bone mineral density; DXA: dual-energy X-ray absorptiometry; NSAID: non-steroid anti-inflammatory drugs; CI: confidence interval; qCT: quantitative computed tomography; OR: odds ratio; BMAD: bone mineral apparent density (volumetric BMD); cDDD: cumulative defined daily dose.

**Table 7:** Characteristics of publications discussing the correlation between clopidogrel and fracture.

	Design	Population Characteristics	Clopidogrel Exposure	Outcome
Jorgensen et al. (2012) [5]	Cohort	77,503 clopidogrel users between 1996 and 2008 in Denmark were recruited, and 232,510 non-users were randomly matched by age and sex as controls.	<0.10, 0.10–0.39, 0.40–0.99, ≥1 DDD	Fracture For clopidogrel, both increased risk of overall fracture and osteoporotic fractures were present, especially in treatment duration of more than 1 year. However, individuals with low exposure to clopidogrel (<0.01 DDD) had a lower risk of fracture than never users.
Jorgensen et al. (2017) [24]	Cohort	77,503 clopidogrel users between 1996 and 2008 in Denmark were recruited, and 232,510 non-users were randomly matched by age and sex as controls. The study end-points were occurrence of stroke, TIA, and occurrence of fracture. 7	<0.10, 0.10–0.39, 0.40–0.79, ≥0.8 DDD	Fracture After adjusting for multiple confounders, clopidogrel use was not associated with increased fracture risk in subjects with ischemic stroke or TIA. In contrast, after adjusting for multiple confounders clopidogrel treatment was associated with a 10–35% reduced risk of fracture

DDD: daily defined dose; TIA: transient ischemic attack.

## Data Availability

The data presented in this study are available on request from the corresponding author.
